# Association between Dietary Inflammatory Index and Metabolic Syndrome in the General Korean Population

**DOI:** 10.3390/nu10050648

**Published:** 2018-05-21

**Authors:** Hye-Young Kim, Jeonghee Lee, Jeongseon Kim

**Affiliations:** 1 Department of Cancer Biomedical Science, Graduate School of Cancer Science and Policy, National Cancer Center, Goyang-si 10408, Korea; hypkim@yongin.ac.kr (H.-Y.K.); jeonghee@ncc.re.kr (J.L.); 2 Department of Foods and Nutrition, Yongin University, Yongin 17092, Korea

**Keywords:** diet, inflammation, metabolic syndrome, adult

## Abstract

Inflammation is thought to be partly responsible for metabolic syndrome (MetS). Recently, dietary inflammatory index (DII) was developed to calculate the overall inflammatory potential of a diet. The objective of this study was to investigate the association between DII and MetS, as well as MetS components, using nationally representative survey data. The study sample consisted of 9291 Korean adults (aged 19–65 years, 3682 men and 5609 women) who participated in the sixth (2013–2015) Korea National Health and Nutrition Examination Survey. DII values were calculated using 24-h dietary recall data. Multivariable-adjusted logistic regression analysis was performed to identify the association between DII and MetS by sex. In the multivariate logistic regression model, the top DII quartile (Q4), was positively associated with MetS prevalence in men (Q4 vs. Q1, OR = 1.40; 95% CI = 1.06–1.85; *p* for linear trend = 0.008) and in postmenopausal women (Q4 vs. Q1, OR = 1.67; 95% CI = 1.15–2.44; *p* for linear trend = 0.008). The top DII quartile was also positively associated with the prevalence of hyperglycemia in men and the prevalence of central obesity in postmenopausal women. Further studies using prospective cohorts are needed to identify the causal relationship between DII and MetS.

## 1. Introduction

Inflammation contributes to the pathophysiology of many chronic diseases, such as obesity, metabolic syndrome (MetS), diabetes, and cardiovascular disease [1,2,3]. Continuous chronic stress exposure leads to chronic low-grade inflammation [4]. Since inflammatory adipokines and cytokines are increased, and anti-inflammatory adipokines and cytokines are reduced, in an inflammatory environment, various metabolic disorders occur over time, resulting in MetS [4,5]. Various factors, such as age, sex, exercise, smoking, and diet are involved in the progression of this inflammatory process [4]. Diet is considered a powerful modulator of low-grade inflammatory conditions because it can affect the balance between pro- and anti-inflammatory cytokines and adipokines [2,4,5].

MetS consists of several factors, including hypertension, hyperglycemia, hypertriglyceridemia, abdominal obesity, and low high-density lipoprotein (HDL) cholesterol [6]. The incidence of MetS has increased globally with obesity and sedentary lifestyles [7]. MetS and its components have become a major public health concern, as they are known risk factors for type 2 diabetes and heart disease [7,8].

The dietary inflammatory index (DII) was recently developed and is used to calculate the overall inflammatory potential of a diet [9]. The DII was developed based on the relationship between various dietary factors and blood inflammatory biomarkers that have been reported in the literature [9]. Several studies have reported a close association between the DII and inflammatory biomarkers [10,11,12,13]. A high DII value is associated with increased high-sensitivity c-reactive protein, interleukin-6, homocysteine, and tumor necrosis factor-α levels [10,11,12,13].

If the inflammatory potential of a diet is high, inflammation-associated MetS may occur easily. In a prospective French study, an inflammatory diet with a high DII value was associated with an increased risk of developing MetS [14]. However, several other prospective and cross-sectional studies from different countries revealed no association between the DII and MetS prevalence [15,16,17,18,19]. Other studies have reported that the DII is related to only some components of MetS [20,21]. In this study, we aimed to investigate the relationship between the DII and MetS in Korean adults using the most recent nationally representative survey data.

## 2. Material and Methods

### 2.1. Study Subjects

This study was conducted based on data from the sixth (2013–2015) Korea National Health and Nutrition Examination Survey (KNHANES). The KNHANES is a nationally representative, cross-sectional survey conducted by the Korea Centers for Disease Control and Prevention (KCDCP) [22]. The KNHANES involves a complex, stratified, multistage probability sampling design. A total of 13525 Korean adults, 19–65 years old, were selected. Among these participants, those who did not respond to the daily dietary survey by the 24-h method were excluded (*n* = 1724). The following participants were also excluded: those who had an energy intake <500 or >5000 kcal/day (*n* = 223); those with incomplete anthropometric data or biochemical data for the diagnosis of MetS (*n* = 1589); and those who did not have the demographic variables or lifestyle data needed for statistical correction (*n* = 698). As a result, 9291 subjects were included in the final analysis. All participants provided written informed consent, and the survey was approved by the Institutional Review Board (IRB) of the KCDCP (2013-07CON-03-4C and 2013-12EXP-03-5C). This study was conducted after research ethics approval was obtained (YIIRB No. 2-1040966-AB-N-01-20-1712-HSR-092-8).

### 2.2. Measurement of Dietary Intake and Other Variables

Food and nutrient intake was assessed from the 24-h dietary recall data. Dietary data were collected from each participant by interviewers trained by the Korea Centers for Disease Control and Prevention (KCDCP). Information on sociodemographic characteristics and health-related variables was obtained during the health interview using a questionnaire. Regarding alcohol consumption, participants were categorized as non-drinkers or ever-drinkers, and for smoking status, as non-smokers (smoked less than 5 packs ever) or smokers. Regular physical activity was defined as at least 30 min per day for 5 days per week at a moderate intensity. To identify education level, the participant was identified as an elementary or middle school graduate, high school graduate, or college graduate.

### 2.3. Measurements of Metabolic Risk Factors

Height and weight were measured to the nearest 0.1 cm and 0.1 kg, respectively; the participants wore light clothing and no shoes. Body mass index (BMI) was calculated as the weight in kilograms divided by the square of the height in meters (kg/m^2^). Waist circumference (cm) was measured at the midpoint between the inferior margin of the last rib and the iliac crest in a horizontal plane.

Blood samples were collected in the morning after the participant had fasted for at least 8 h. Fasting plasma glucose, triglyceride, and HDL cholesterol levels (mg/dL) were measured enzymatically using a Hitachi automatic analyzer 7600 (Hitachi, Tokyo, Japan) in central, certified laboratories. Blood pressure was measured with a Baumanometer mercury sphygmomanometer (W.A. Baum, Copiague, NY, USA). Three systolic blood pressure (SBP) and diastolic blood pressure (DBP) readings were recorded, and the average of the last two readings was used for data analysis.

### 2.4. Definition of Metabolic Syndrome

MetS was diagnosed, based on the Modified National Cholesterol Education Program Adult Treatment Panel III (NCEP-ATP III) and the obesity guidelines of the Obesity Society of Korea, as ≥3 of any of the following [23,24]: (1) abdominal obesity (WC ≥90 cm for men or ≥85 cm for women); (2) high blood pressure (SBP/DBP ≥130/85 mmHg or the use of antihypertensive medication); (3) hypertriglyceridemia (triglyceride level ≥150 mg/dL); (4) hyperglycemia (fasting plasma glucose ≥100 mg/dL or current use of insulin or oral hypoglycemia medication, or a physician’s diagnosis); and (5) low HDL-cholesterol level (<40 mg/dL in men or <50 mg/dL in women).

### 2.5. Calculation of Dietary Inflammation Index

The inflammatory potential of the diet was calculated using the DII developed by Shivappa et al. [9]. The DII development details [9] and construct validation [10,11,12,13] have been previously described. The 24-h dietary recall data were used to calculate the *z*-score for each of the food parameters for each individual. The global means and standard deviations of the food and nutrient intakes collected from the 11 nations, including Korea, were used to calculate the *z*-score [9]. The *z*-score was then converted to a percentile and centered by doubling the value and subtracting 1. The centered percentile value was multiplied by the respective inflammatory effect score to obtain the food parameter-specific DII score [9]. The overall DII score was calculated as the sum of all the available food parameter-specific DII scores. In this study, data on 23 of the original 45 DII food parameters were available and were used for DII calculation. These parameters included seven proinflammatory components (energy, carbohydrates, protein, total fat, saturated fatty acids, cholesterol, and iron) and 16 anti-inflammatory components (monounsaturated fatty acids (MUFA), polyunsaturated fatty acids (PUFA), n-3 fatty acids, n-6 fatty acids, fiber, vitamin A, β-carotene, thiamin, riboflavin, niacin, vitamin C, garlic, ginger, onion, tea, and pepper). Parameters not yet available in the KNHANES data due to an incomplete food database or very low intake were not included in the DII calculation. Energy-adjusted values, which were obtained using the residual method, were used [25]. A low DII value indicates that the diet has low inflammatory potential.

### 2.6. Statistical Analyses

All statistical analysis accounted for the complex sampling designs and used appropriate sampling weights, using PROC SURVEY in the Statistical Analysis Systems (SAS) program to make estimates of the entire Korean adult population from the representative survey sample. Categorical variables were expressed as frequencies and percentages, and continuous variables were expressed as the means and standard errors. To determine differences in the variables according to sex, *t*-tests were used for continuous variables, and the chi-square test, for categorical variables.

The DII was categorized into quartiles according to the distribution of the subjects by sex. Differences across the DII quartiles by sex were determined using chi-square tests (categorical variables) or ANOVA (continuous variables). Logistic regression analysis was performed to determine odds ratios (ORs) and 95% confidence intervals (CIs) for MetS and its components according to the DII quartiles. The bottom DII quartile (Q1) was used as a reference category. In the multivariable-adjusted model, age, body mass index (BMI), education, smoking, alcohol consumption, physical activity, and total energy intake were considered as potential confounders. Linear trends across the DII were tested by assigning the median value of the category to each participant and modeling this value as a continuous variable. A stratified analysis using menopausal status was performed to examine the possibility of effect modification by menopausal status. SAS 9.4 software (SAS Institute, Inc., Cary, NC, USA) was used to perform the calculations; a 2-sided *p* value less than 0.05 was considered statistically significant.

## 3. Results

The characteristics of the study participants are presented in Table 1. A total of 9291 subjects (3682 men and 5609 women) were studied with a mean age of 41.3 years. The BMI (kg/m^2^) of the subjects was 23.7, and the mean energy intake was 2136.2 kcal. Men were more likely than women to consume alcohol, smoke, and engage in aerobic activity. The mean DII score, waist circumference, systolic and diastolic blood pressure, blood triglyceride and fasting glucose levels were higher in men than in women. However, blood HDL cholesterol levels were higher in women than in men. The prevalence of MetS was higher in men (26.5%) than in women (14.9%).

Lifestyle characteristics and nutrient and food intake according to the DII quartiles stratified by sex are shown in Table 2. In both men and women, subjects with high DII values were younger, more likely to drink alcohol, and had higher energy intake levels than those with low DII values. In men, subjects with high DII values had a lower BMI than those with low DII values. In women, subjects with high DII values were more likely to be current smokers than those with low DII values. Nutrient and food intake trends according to the DII quartile were similar for both sexes. Compared with those in the bottom DII quartile, subjects in the top DII quartile had a higher proportion of energy intake from carbohydrates and a lower proportion of energy intake from protein. Additionally, subjects in the top DII quartile had a higher intake of saturated fat, MUFA, and cholesterol but a lower intake of PUFA and n-3 fatty acids. Subjects with high DII values also had significantly reduced fiber, iron, and vitamin intake. Garlic, ginger, onion, tea, and pepper intake were also significantly reduced in subjects with high DII values.

Table 3 presents the ORs of MetS and its components according to DII quartiles. In the crude model, DII was not associated with MetS prevalence in either men or women. In the multivariable-adjusted logistic regression model, the top DII quartile (Q4) was positively associated with MetS prevalence only in men (Q4 vs. Q1, OR = 1.40; 95% CI = 1.06–1.85; *p* for linear trend = 0.008) after controlling for age, BMI, education, alcohol consumption, smoking, physical activity, and total calorie intake. Among men, the top DII quartile was also positively associated with the prevalence of hyperglycemia (Q4 vs. Q1, OR = 1.30; 95% CI = 1.02–1.65; p for linear trend = 0.038).

**Figure 1:**
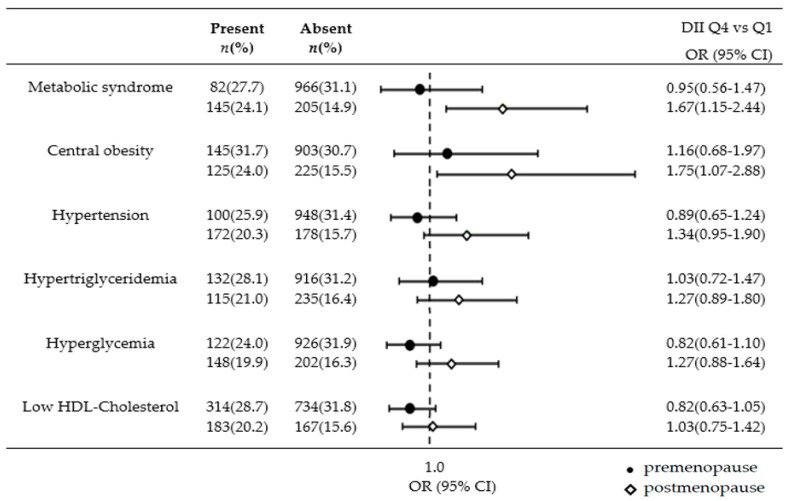
Odds ratio of metabolic syndrome risk factors by menopausal status of women by DII quartiles. DII: dietary inflammatory index; OR: odds ratio; CI: confidence interval. All analyses accounted for the complex sampling design and appropriate sampling weights of the national survey. Data were adjusted for age, BMI, education, alcohol consumption, smoking, physical activity, and total calorie intake.

When women were stratified by menopausal status, an association between the DII and MetS prevalence was identified in postmenopausal women (Figure 1). The top DII quartile (Q4) was positively associated with MetS prevalence (Q4 vs. Q1, OR = 1.67; 95% CI = 1.15–2.44; p for linear trend = 0.008) and central obesity prevalence (Q4 vs. Q1, OR = 1.75; 95% CI = 1.07–2.88; p for linear trend = 0.070) in postmenopausal women after controlling for confounding factors.

## 4. Discussion

In this study, we used the nationally representative KNHANES data to investigate the association between the DII and MetS in Korean adults. The DII was significantly associated with MetS prevalence in men and postmenopausal women. The DII was also positively associated with the prevalence of hyperglycemia in men and central obesity in postmenopausal women. 

Several studies have examined the association between DII and MetS, and the results to date have been inconsistent. The prospective Supplementation en Vitamines et Mineraux AntioXydants (SUVIMAX) cohort study in France [14] examined the association between the DII and MetS outcomes in 3726 subjects. The authors found an increased risk of developing MetS with high DII values (OR compared Q4 to Q1 was 1.39, 95% CI = 1.01–1.92) after an average follow-up of 12.4 years. Another prospective study of a Spanish cohort of university graduates (SUN) assessed the association between different dietary indexes and MetS incidence after a median follow-up of 8.3 years (*n* = 6851) [15]. They found that the Pro-Vegetarian Diet was significantly associated with a lower risk of developing MetS, but no significant association was found between the DII and MetS. Several other cross-sectional studies, including the Polish-Norwegian (PONS) study (*n* = 3862), Buffalo Cardio-Metabolic Occupational Police Stress (BCOPS) study (*n* = 464), Luxembourg study (*n* = 1352), and Lebanese study (*n* = 331), also assessed the association between the DII and MetS and found no significant association [16,17,18,19]. Overall, only one longitudinal study (SUVIMAX) and one cross-sectional study (the present work) have found a positive association between DII and MetS; no other studies have found a link between DII and MetS.

The inconsistent results regarding the association between the DII and MetS might be due to several reasons, such as differences in population, diet, study design, sample size, and age [14,15,16,17,18,19]. It is also possible that the inconsistent results among studies may be due, in part, to different dietary data collection methods and available food parameters used to calculate DII. The present study used 24-h dietary recall of the participants to collect dietary data and used 23 dietary parameters that included anti-inflammatory foods, such as garlic, onion, pepper, and tea, as well as nutrient parameters to calculate DII. Other dietary parameters were not used in this study because of an incomplete food database or very low intake. The SUVIMAX study [14] used 24-h dietary records to obtain 36 food parameters, including anti-inflammatory foods and flavonoids. Other MetS studies [15,16,17,18,19], on the other hand, obtained 22–28 parameters in a food frequency questionnaire and did not include anti-inflammatory spice parameters.

Anti-inflammatory foods, such as pepper, onion, garlic, and tea, have been consumed in large quantities for a long time in Korea. In this study, the lower DII quartile (Q1) consumed much more pepper, garlic, onion, and tea than the upper DII quartile (Q4). Pepper (capsaicin) consumption has been linked to cardiovascular health by improving endothelial function, reducing body fat, and increasing the resistance of lipoprotein to oxidation [26,27]. The administration of aged garlic extract increased adiponectin, an anti-inflammatory adipokine with cardioprotective properties [28]. Onion was effective as a cholesterol-lowering food agent [29]. Green tea also increased adiponectin and decreased pro-inflammatory serum amyloid alpha [30,31]. The high intake of these anti-inflammatory foods may have increased the synergy of anti-inflammatory potential to inhibit the development of MetS. The contribution of each anti-inflammatory food to MetS development has not been investigated, and further research is needed in the future.

The Mediterranean diet and DASH diet have been reported to lower MetS prevalence [8,32]. Components of these healthy dietary patterns that may contribute to reducing MetS development include whole grains, fruits and vegetables, dairy products, and n-3 fatty acids [33,34,35]. In our study, fiber intake, vitamin A, vitamin C, n-3 fatty acids, and PUFA were included as DII components, and intake of these nutrients was inadequate in high inflammatory quartiles. Thus, some of the effects that the DII may have on MetS development are associated with factors that decrease MetS in a healthy eating pattern.

The association between the DII and MetS was more pronounced in postmenopausal women than in premenopausal women. After menopause, the lack of estrogen has been shown to alter fat metabolism and promote MetS [36]. Thus, dietary inflammatory potential appears to have an increased impact on postmenopausal women who are prone to developing MetS.

The association between the DII and metabolic risk factors has been examined in several studies. In the prospective SUVIMAX study [15], high DII scores were associated with increased triglyceride levels and blood pressure and reduced HDL cholesterol levels. In the cross-sectional BCOPS study [17], the glucose intolerance component was affected by a pro-inflammatory diet. In the PREvención con DIeta MEDiterránea (PREDIMED) cross-sectional study in Spain [20], the DII was associated with abdominal obesity in women, even after controlling for adherence to the Mediterranean diet, showing a clear relationship between dietary inflammation and abdominal obesity. In a longitudinal study of Australian adults [21], more inflammatory diets were associated with an increased risk of hypertension, suggesting that inflammation adversely affects blood vessels and kidneys, leading to hypertension [37]. In the Luxembourg and Lebanese studies, however, no association was observed between the DII and metabolic risk factors [18,19]. The association between DII and metabolic risk factors varied from study to study, and further studies are needed to form a clear conclusion.

In the present study, we found a sex and age difference in the effects of a pro-inflammatory diet on metabolic risk factors. The DII was associated with hyperglycemia in men. The DII was also associated with central obesity in postmenopausal women, but not in premenopausal women. The reasons why the DII has different effects on metabolic risk factors depending on sex and age are unclear. The first possible reason is that postmenopausal women have more body fat than premenopausal women, and this body fat, especially abdominal fat, is thought to be involved in producing many inflammatory markers in the body [38]. Second, sex hormones may influence the level of inflammatory markers and the regulation of metabolic risk factors [39,40]. 

The strengths of this study are as follows: The KNHANES is a nationally representative survey; the sample size (*n* = 9291) of this study was large. However, the following are some limitations of our study. First, the cross-sectional design of this study essentially limited causal inferences. Second, we used data from a single 24-h recall, which may not represent the usual intake of the subject. Third, only 23 out of 45 original dietary inflammatory parameters were available to calculate the DII. Fourth, we did not have information on specific hypertriglyceridemia and HDL-cholesterol medications taken by the subjects to include in the MetS criteria. Fifth, the associations between the DII and MetS found in this study cannot be generalized to populations with very different dietary styles.

## 5. Conclusions

The DII was significantly associated with MetS prevalence after controlling for various confounding factors in Korean men and postmenopausal women. The DII was also positively associated with the prevalence of hyperglycemia in men and central obesity in postmenopausal women. Further prospective cohort studies should be conducted before a causal association between the DII and MetS can be concluded.

## Figures and Tables

**Table 1:** General characteristics of the subjects. ^1.^

Characteristics	Total (*n* = 9291)	Men (*n* = 3682)	Women (*n* = 5609)	*p*-Value ^2^
Age (years)	41.3 ± 0.2	40.9 ± 0.2	41.8 ± 0.2	0.002
BMI (body mass index, kg/m^2^)	23.7 ± 0.0	24.5 ± 0.1	23.0 ± 0.1	<0.001
Energy intake (kcal/day)	2136.2 ± 11.4	2463.7 ± 17.2	1810.3 ± 10.6	<0.001
Education				
≤Middle school	1907 (16.0)	625 (12.8)	1282 (19.2)	<0.001
High school	3675 (41.7)	1474 (42.8)	2201 (40.6)	
≥University	3709 (42.3)	1583 (44.4)	2126 (40.3)	
Alcohol consumption	8525 (93.2)	3556 (96.6)	4969 (89.9)	<0.001
Smoking	3153 (39.4)	2666 (69.8)	487 (9.2)	<0.001
Physical activity	3883 (45.1)	1659 (48.1)	2224 (42.0)	<0.001
Metabolic syndrome	2009 (20.7)	1043 (26.5)	966 (14.9)	<0.001
Waist circumference (cm) ^3^	80.7 ± 0.1	85.4 ± 0.2	76.9 ± 0.2	<0.001
Systolic blood pressure (mmHg) ^3^	114.3 ± 0.2	118.9 ± 0.3	111.7 ± 0.2	<0.001
Diastolic blood pressure (mmHg) ^3^	75.6 ± 0.2	79.2 ± 0.2	73.1 ± 0.2	<0.001
Triglyceride (mg/dL) ^3^	135.5 ± 1.5	170.5 ± 2.6	108.5 ± 1.1	<0.001
Fasting glucose (mg/dL) ^3^	97.3 ± 0.3	101.2 ± 0.4	95.9 ± 0.3	<0.001
HDL cholesterol (mg/dL) ^3^	51.5 ± 0.1	47.1 ± 0.2	55.2 ± 0.2	<0.001
Dietary inflammatory index score	0.56 ± 0.02	0.88 ± 0.03	0.25 ± 0.03	<0.001

All analyses accounted for the complex sampling design and appropriate sampling weights of the national survey. ^1^ Data are expressed as the number of subjects for each category (weighted percentage) or means ± SE (standard error). ^2^ All *p*-values represent differences between men and women, *p*-values are from the chi-square test for categorical variables and t-test for continuous variables. ^3^ Means ± SE were adjusted for age and sex.

**Table 2:** Characteristics and nutrient and food intake of the subjects according to DII quartiles.

	Men					Women				
DII Quartile	Q1 <−0.16 (*n* = 920)	Q2 −0.16 –<0.91 (*n* = 921)	Q3 0.91 –< 1.89 (*n* = 920)	Q4 ≥1.89 (*n* = 921)	*p*-Value ^1^	Q1 <−0.85 (*n* = 1403)	Q2 −0.85 –< 0.20 (*n* = 1402)	Q3 0.20 –< 1.28 (*n* = 1402)	Q4 ≥1.28 (*n* = 1402)	*p*-Value
Age (years)	43.8 ± 0.5	41.8 ± 0.4	40.6 ± 0.5	37.7 ± 0.5	<0.001	45.9 ± 0.3	43.0 ± 0.4	41.3 ± 0.4	37.3 ± 0.4	<0.001
BMI (kg/m^2^)	24.9 ± 0.1	24.4 ± 0.1	24.5 ± 0.1	24.2 ± 0.1	0.001	23.1 ± 0.1	23.0 ± 0.1	22.9 ± 0.1	22.8 ± 0.1	0.205
Education level										
≤Middle school	169(14.3)	147(12.3)	165(13.6)	144(11.1)	<0.001	335(21.2)	350(20.6)	326(19.9)	271(15.4)	0.001
High school	311(35.5)	351(40.0)	384(44.5)	428(50.3)		580(42.7)	528(38.3)	531(39.0)	562(42.2)	
≥University	440(50.2)	423(47.7)	371(41.9)	349(38.6)		488(36.2)	524(41.0)	545(41.1)	569(42.4)	
Alcohol consumption (yes)	875(95.2)	875(95.2)	897(97.7)	893(97.1)	0.048	1218(88.1)	1233(89.4)	1248(90.1)	1270(91.7)	0.029
Smoking (yes)	660(69.2)	651(68.3)	673(71.3)	682(70.4)	0.605	80(6.4)	107(8.4)	130(9.7)	170(11.9)	<0.001
Physical activity (yes)	402(46.1)	420(48.3)	413(47.3)	424(50.6)	0.296	605(45.3)	575(43.0)	498(37.7)	546(42.2)	0.005
Energy (kcal)	2058.4 ± 28.1	2425.2 ± 31.3	2585.4 ± 31.2	2745.6 ± 32.4	<0.001	1587.9 ± 17.3	1745.5 ± 19.4	1872.4 ± 20.8	2006.9 ± 23.6	<0.001
% carbohydrates	64.7 ± 0.4	65.6 ± 0.4	66.3 ± 0.4	67.1 ± 0.4	<0.001	63.9 ± 0.3	66.6 ± 0.3	66.3 ± 0.3	66.1 ± 0.3	<0.001
% protein	15.3 ± 0.1	14.5 ± 0.1	13.6 ± 0.1	12.6 ± 0.1	<0.001	15.3 ± 0.1	13.9 ± 0.1	13.7 ± 0.1	12.9 ± 0.1	<0.001
% fat	20.0 ± 0.3	19.9 ± 0.3	20.1 ± 0.3	20.2 ± 0.3	0.936	20.8 ± 0.3	19.5 ± 0.2	20.0 ± 0.3	21.0 ± 0.3	<0.001
Saturated fat (g)	12.2 ± 0.3	15.3 ± 0.4	17.5 ± 0.4	20.6 ± 0.5	<0.001	9.5 ± 0.2	10.7 ± 0.2	12.4 ± 0.3	15.7 ± 0.3	<0.001
MUFA (g)	14.4 ± 0.4	17.8 ± 0.5	19.2 ± 0.5	21.4 ± 0.6	<0.001	11.5 ± 0.3	12.2 ± 0.3	13.6 ± 0.3	15.8 ± 0.4	<0.001
PUFA (g)	14.0 ± 0.4	14.9 ± 0.4	14.2 ± 0.4	12.4 ± 0.3	<0.001	11.6 ± 0.3	10.4 ± 0.2	10.8 ± 0.3	9.5 ± 0.2	<0.001
n-3 Fatty acids (g)	2.3 ± 0.1	2.1 ± 0.1	1.8 ± 0.1	1.4 ± 0.0	<0.001	2.0 ± 0.1	1.5 ± 0.0	1.5 ± 0.0	1.1 ± 0.0	<0.001
n-6 Fatty acids (g)	11.8 ± 0.4	12.9 ± 0.3	12.5 ± 0.3	11.1 ± 0.3	<0.001	9.6 ± 0.2	8.9 ± 0.2	9.4 ± 0.3	8.4 ± 0.2	<0.001
Cholesterol (mg)	276.8 ± 9.1	311.0 ± 10.0	323.4 ± 10.0	339.2 ± 10.4	<0.001	212.8 ± 5.9	221.6 ± 6.8	246.5 ± 6.6	268.7 ± 7.0	<0.001
Fiber (g)	31.3 ± 0.6	29.3 ± 0.5	25.5 ± 0.4	18.5 ± 0.3	<0.001	27.8 ± 0.4	24.8 ± 0.4	21.9 ± 0.3	16.7 ± 0.4	<0.001
Fe (mg)	22.5 ± 1.2	22.5 ± 1.2	19.7 ± 0.4	17.6 ± 0.4	<0.001	17.2 ± 0.4	16.1 ± 0.3	15.3 ± 0.2	13.8 ± 0.3	<0.001
Vitamin A (mg)	1338.8 ± 64.5	891.1 ± 26.9	688.2 ± 32.7	486.2 ± 11.3	<0.001	1180.8 ± 40.4	723.8 ± 23.1	545.4 ± 15.8	401.1 ± 8.6	<0.001
Carotene (μg)	6960.6 ± 325	4278.7 ± 125	2985.1 ± 72.4	1958.3 ± 54.1	<0.001	6324.1 ± 229.1	3568.4 ± 116.4	2418.6 ± 68.9	1527.1 ± 37.2	<0.001
Vitamin B_1_ (mg)	2.4 ± 0.0	2.5 ± 0.0	2.4 ± 0.0	2.3 ± 0.0	0.002	1.9 ± 0.0	1.9 ± 0.0	1.9 ± 0.0	1.7 ± 0.0	<0.001
Vitamin B_2_ (mg)	1.6 ± 0.0	1.7 ± 0.0	1.6 ± 0.0	1.6 ± 0.0	0.603	1.3 ± 0.0	1.3 ± 0.0	1.3 ± 0.0	1.3 ± 0.0	0.043
Niacin (mg)	19.7 ± 0.4	20.6 ± 0.4	20.3 ± 0.4	18.8 ± 0.4	0.004	15.7 ± 0.2	15 ± 0.2	15.3 ± 0.2	14.1 ± 0.2	<0.001
Vitamin C (mg)	137.7 ± 4.3	112.8 ± 4.3	85.0 ± 3.9	51.1 ± 1.9	<0.001	146.1 ± 4.1	131.4 ± 4.9	102.5 ± 3.9	57.7 ± 2.3	<0.001
Garlic (g)	10.5 ± 0.7	7.8 ± 0.4	7.3 ± 0.5	4.9 ± 0.3	<0.001	6.8 ± 0.3	5.8 ± 0.3	4.6 ± 0.3	2.6 ± 0.1	<0.001
Ginger (g)	0.6 ± 0.0	0.8 ± 0.2	0.6 ± 0.1	0.4 ± 0.0	0.001	0.4 ± 0.1	0.4 ± 0.0	0.3 ± 0.0	0.2 ± 0.0	0.006
Onion (g)	39.4 ± 1.7	38.4 ± 1.7	36.7 ± 2.6	26.7 ± 1.3	<0.001	31.5 ± 1.4	27.4 ± 1.1	22.6 ± 1.2	18.7 ± 1.1	<0.001
Green/black tea (g)	49.4 ± 7.0	20.6 ± 3.8	16.7 ± 5.8	6.5 ± 3.5	<0.001	53.9 ± 7.9	23.3 ± 3.9	15.5 ± 3.0	4.0 ± 1.5	<0.001
Pepper (g)	53.5 ± 2.8	44.2 ± 2.0	33.2 ± 1.3	21.7 ± 1.1	<0.001	43 ± 2.0	30.1 ± 1.2	22.8 ± 1.2	12.5 ± 0.6	<0.001

DII: dietary inflammatory index; MUFA: monounsaturated fatty acids; PUFA: polyunsaturated fatty acids. All analyses accounted for the complex sampling design and appropriate sampling weights of the national survey. Data are expressed as the number of subjects for each category (weighted percentage) or means ± SE (standard error). ^1^ *p*-values are from ANOVA test.

**Table 3:** Odds ratio of metabolic syndrome and its components by DII quartiles.

	Men				Women			
DII Quartiles ^1^	Present *n* (%)	Absent *n* (%)	Crude OR (95% CI)	Fully Adjusted OR ^2^ (95% CI)	Present *n* (%)	Absent *n* (%)	Crude OR (95% CI)	Fully Adjusted OR ^2^ (95% CI)
Metabolic syndrome								
Q1	283(26.0)	637(23.1)	1.00(ref.)	1.00(ref.)	261(26.4)	1142(23.2)	1.00(ref.)	1.00(ref.)
Q2	244(22.5)	677(25.2)	0.80(0.64–0.99)	0.98(0.76–1.27)	240(23.8)	1162(24.3)	0.86(0.69–1.07)	0.88(0.68–1.26)
Q3	272(25.9)	648(24.2)	0.95(0.76–1.20)	1.25(0.96–1.64)	238(24.2)	1164(25.4)	0.84(0.67–1.05)	1.02(0.78–1.34)
Q4	244(25.6)	677(27.5)	0.83(0.66–1.04)	1.40(1.06–1.85)	227(25.5)	1175(27.0)	0.83(0.66–1.04)	1.22(0.91–1.64)
*p* for trend ^3^			0.232	0.008			0.120	0.180
Central obesity								
Q1	278(27.0)	642(22.7)	1.00(ref.)	1.00(ref.)	239(22.2)	1164(24.0)	1.00(ref.)	1.00(ref.)
Q2	233(22.5)	688(25.2)	0.75(0.60–0.94)	0.91(0.64–1.31)	273(24.8)	1129(24.1)	1.11(0.91–1.36)	1.19(0.86–1.64)
Q3	274(26.8)	646(23.9)	0.94(0.75–1.19)	1.28(0.89–1.85)	262(24.8)	1140(25.3)	1.06(0.85–1.33)	1.28(0.90–1.83)
Q4	225(23.7)	696(28.2)	0.71(0.56–0.89)	1.07(0.72–1.61)	270(28.2)	1132(26.5)	1.15(0.93–1.42)	1.35(0.94–1.94)
*p* for trend ^3^			0.020	0.445			0.282	0.094
Hypertension								
Q1	389(26.5)	531(22.4)	1.00(ref.)	1.00(ref.)	397(29.4)	1006(22.2)	1.00(ref.)	1.00(ref.)
Q2	335(22.7)	586(25.5)	0.75(0.61–0.93)	0.85(0.68–1.07)	351(25.1)	1051(24.0)	0.79(0.65–0.96)	0.92(0.75–1.14)
Q3	360(24.9)	560(24.6)	0.86(0.69–1.06)	1.00(0.79–1.27)	313(23.0)	1089(25.8)	0.67(0.56–0.82)	0.89(0.73–1.10)
Q4	345(25.9)	576(27.6)	0.79(0.64–0.99)	1.14(0.88–1.46)	274(22.4)	1128(28.0)	0.61(0.50–0.74)	1.10(0.87–1.38)
*p* for trend ^3^			0.086	0.217			<0.001	0.616
Hypertriglyceridemia								
Q1	366(24.2)	554(23.6)	1.00(ref.)	1.00(ref.)	270(24.7)	1133(23.5)	1.00(ref.)	1.00(ref.)
Q2	363(23.8)	558(25.0)	0.93(0.76–1.14)	1.06(0.85–1.33)	270(24.1)	1132(24.3)	0.95(0.76–1.18)	1.01(0.81–1.27)
Q3	392(25.6)	528(24.1)	1.04(0.84–1.28)	1.19(0.95–1.49)	273(26.4)	1129(25.0)	1.01(0.82–1.24)	1.14(0.91–1.43)
Q4	368(26.5)	553(27.3)	0.95(0.77–1.17)	1.22(0.97–1.53)	247(24.7)	1155(27.3)	0.86(0.69–1.08)	1.07(0.84–1.38)
*p* for trend ^3^			0.848	0.061			0.272	0.418
Hyperglycemia								
Q1	384(26.9)	536(22.3)	1.00(ref.)	1.00(ref.)	371(27.6)	1032(22.7)	1.00(ref.)	1.00(ref.)
Q2	329(23.2)	592(25.2)	0.76(0.62–0.94)	0.90(0.71–1.14)	319(24.7)	1083(24.1)	0.84(0.69–1.02)	0.95(0.77–1.18)
Q3	338(23.6)	582925.2)	0.78(0.62–0.97)	0.97(0.76–1.24)	331(25.9)	1071(25.1)	0.85(0.71–1.01)	1.08(0.88–1.32)
Q4	333(26.2)	588(27.3)	0.79(0.65–0.97)	1.30(1.02–1.65)	271(21.8)	1131(28.1)	0.64(0.52–0.77)	0.95(0.77–1.18)
*p* for trend ^3^			0.037	0.038			<0.001	0.914
Low HDL-cholesterol								
Q1	250(26.5)	670(23.0)	1.00(ref.)	1.00(ref.)	586(27.2)	817(21.8)	1.00(ref.)	1.00(ref.)
Q2	235(24.9)	686(24.4)	0.89(0.70–1.12)	1.05(0.82–1.34)	514(23.5)	888(24.6)	0.76(0.65–0.90)	0.79(0.67–0.94)
Q3	231(26.1)	689(24.2)	0.94(0.75–1.17)	1.14(0.90–1.45)	495(23.9)	907(26.0)	0.74(0.62–0.88)	0.78(0.65–0.94)
Q4	186(22.5)	735(28.3)	0.69(0.55–0.87)	0.93(0.72–1.21)	497(25.4)	905(27.6)	0.74(0.62–0.87)	0.85(0.71–1.04)
*p* for trend ^3^			0.004	0.788			0.001	0.113

DII: dietary inflammatory index; OR: odds ratio; CI: confidence interval; ref., reference. All analyses accounted for the complex sampling design and appropriate sampling weights of the national survey. ^1^ The DII scores were categorized into quartiles for men (Q1: <−0.16, Q2: −0.16–<0.91, Q3: 0.91–<1.89, Q4: ≥1.89) and for women (Q1: <−0.85, Q2:-0.85–<0.20, Q3: 0.20–<1.28, Q4: ≥1.28). ^2^ Adjusted for age, BMI, education, alcohol consumption, smoking, physical activity, and total calorie intake. ^3^ *p* for linear trend was calculated using the median value of each quartile category as a continuous variable.
